# Compass: Clinical Evaluation of a New Instrument for the Diagnosis of Glaucoma

**DOI:** 10.1371/journal.pone.0122157

**Published:** 2015-03-25

**Authors:** Luca Rossetti, Maurizio Digiuni, Alberto Rosso, Roberta Riva, Giuliano Barbaro, Michael K. Smolek, Nicola Orzalesi, Stefano De Cilla’, Alessandro Autelitano, Paolo Fogagnolo

**Affiliations:** 1 Eye Clinic, Department of Medicine, Surgery and Odontoiatry, Ospedale San Paolo, University of Milan, Milan Italy; 2 CenterVue, Padova, Italy; 3 CLEVER Eye Institute, Slidell, Louisiana, United States of America; 4 Unit of Ophthalmology, Ospedale Maggiore della Carità, Novara, Italy; Bascom Palmer Eye Institute, University of Miami School of Medicine;, UNITED STATES

## Abstract

**Aims:**

To evaluate Compass, a new instrument for glaucoma screening and diagnosis that combines scanning ophthalmoscopy, automated perimetry, and eye tracking.

**Materials and Methods:**

A total of 320 human subjects (200 normal, 120 with glaucoma) underwent full ophthalmological evaluation and perimetric evaluation using the Humphrey SITA standard 24° test (HFA), and the Compass test that consisted of a full-threshold program on the central 24° with a photograph of the central 30° of the retina. A subgroup of normal subjects and glaucoma patients underwent a second Compass test during the same day in order to study test-retest variability. After exclusion of 30 patients due to protocol rules, a database was created to compare the Compass to the HFA, and to evaluate retinal image quality and fixation stability.

**Results:**

The difference in mean sensitivity between Compass and HFA was -1.02 ± 1.55 dB in normal subjects (p<0.001) and -1.01 ± 2.81 dB in glaucoma (p<0.001). Repeatability SD for the average sensitivity was 1.53 for normal subjects and 1.84 for glaucoma. Test time with the Compass was 634±96 s (607±78 for normals, 678±108 for glaucoma). Compass analysis showed the percentage of fixation within the central 1° was 86.6% in normal subjects, and 79.3% in glaucoma patients. Color image quality was sufficient for diagnostic use in >65% of cases; Image-based diagnosis was in accordance with the initial diagnosis in 85% of the subjects.

**Conclusions:**

Based on preliminary results, Compass showed useful diagnostic characteristics for the study of glaucoma, and combined morphological information with functional data.

## Introduction

Glaucoma is the leading cause of irreversible blindness with a prevalence of 3.5% in the population aged 40–80 years. In 2013, the disease affected 64.3 million people worldwide, and is expected to increase to 76 million in 2020 and 111.8 million by 2040 [1].

Due to the lack of symptoms up to advanced stages and to the limitations of world-wide diagnostic resources and information (so that a high number of subjects are very rarely seen by ophthalmologists in the course of their life), the current undetection rate for the disease is about 50%, even in so-called “developed” countries [2–3].

Currently, the diagnosis of glaucoma relies on the assessment of intraocular pressure (IOP) and its confounding factors, as well as specific changes occurring in the visual field (VF), the optic nerve head (ONH), and the retinal nerve fiber layer (RNFL).

VF is studied by means of automated perimetry; the most common perimeter used by ophthalmologists is the Humphrey Field Analyzer (HFA; Zeiss Humphrey Systems, Dublin, Ca, USA). Perimetry is commonly considered the diagnostic gold-standard for glaucoma progression, as it is a reliable test with a very accurate database, which has been validated over many decades of clinical use. In addition, it enables the detection of small glaucomatous changes, which directly impact the visual function of patients. Yet, perimetry is limited by low diagnostic sensitivity in early stages of the disease.[4], and being a psychophysical test, it is also influenced by learning, fatigue, psychophysical status, as well as eye movements. [5]

The use of microperimetry [6], of more accurate perimetric grids, [6] unconventional perimetry [7] and high-tech instruments to assess the thickness of the ONH and RNFL (in particular by means of Optical Coherence Tomography, OCT) [8] may be helpful in early diagnosis. Recent studies indicate that integration of morphological and functional data could strongly enhance glaucoma diagnostic ability [8–9]. The study of fixation may be also of interest for two reasons: it affects the quality of VF tests, and it is abnormal in both early and advanced glaucoma stages compared with controls. [10–11]. Finally, telemedicine could provide a valid help in diagnosing glaucoma on subjects who do not attend regular eye visits. [12]

The aim of this study was to evaluate a new instrument, called Compass (CenterVue, Padova, Italy), which is a fully automated device consisting in a scanning ophthalmoscope (collecting live infrared images of the fundus and color images of the posterior pole without pupil dilation) combined with an automated perimeter and an eye tracker (allowing the study of fixation stability and the accurate presentation of stimuli at predefined retinal locations, thanks to the active compensation of eye movements / fixation losses). Being Compass data easily accessible via Internet, the instrument may play a role in teleglaucoma. For this study we used a 24°, full-threshold perimetric strategy; customized and adaptive perimetric grids are being developed to increase the diagnostic accuracy and reduce test time.

## Methods

This study was conducted at the Eye Clinic of San Paolo Hospital, University of Milan, Italy, from November 2013 to June 2014. It was approved by the “San Paolo Hospital Ethics Committee” (n. 734 of July 30th, 2013—Studio GSD 2013). It followed the requirements of the Helsinki declaration, the guidelines on Good Clinical Practice (GCP). and of the International Conference on Harmonization (ICH). All participants gave their written informed consent.

Study objectives were:

- to compare perimetric data measured by Compass and HFA in patients with glaucoma and normal subjects;
- to generate a first version of a normative database for Compass;
- to measure test-retest variability of Compass perimetric data;
- to evaluate the quality of images of the retina obtained by Compass;
- to explore fixation stability in normal and glaucoma subjects.

The study protocol was open label and cross-sectional. It consisted of one visit to verify inclusion and exclusion criteria, and to then perform the study tests. Examinations consisted of full ophthalmological evaluation; perimetric demonstration in patients inexperienced with perimetry; one HFA test using Standard Swedish Interactive Threshold Algorithm (SITA) standard strategy over central 24°; one Compass test using a conventional full threshold strategy (4–2 staircase, modeled on accelerated stochastic approximation) on the central 24°; a photograph of the central 30° (radius) of the retina with Compass; RNFL OCT evaluation; and red-free and infrared imaging of the retina and optic nerve head. A subgroup of normal subjects and glaucoma patients underwent a second Compass test during the same day with the same operator in order to study test-retest variability.

The study was conducted on both normal subjects and glaucoma patients, on one eye per patient, chosen at random. The sequence of perimetric tests was randomized. A 30-minute interval was observed between any two tests. In the case of an unreliable test (fixation losses, or false positive or false negative > 30%), an attempt was made to repeat the test. if the second test was also unreliable, the patient was excluded from the study.

Inclusion criteria for normal subjects were: age between 20 and 80 years; best-corrected decimal visual acuity >0.8 (for subjects <50 years old) or >0.6 (over 50) in both eyes; spherical refraction within ±5D; astigmatism within ±2D; normal visual field in both eyes (normal mean deviation, MD, and pattern standard deviation, PSD); normal appearance of the optic disc in both eyes; and an IOP ≤ 21 mmHg in both eyes. Inclusion criteria for glaucoma subjects were: age between 20 and 80 years; best-corrected visual acuity >0.8 (for subjects <50 years old) or >0.6 (over 50) in both eyes; spherical refraction within ±5D; astigmatism within ±2D; the appearance of a glaucomatous optic disc in both eyes (a diffuse neuroretinal rim narrowing with concentric enlargement of the optic cup or localized notching, or both); and IOP>22 mmHg in both eyes in subjects without treatment or <21 mmHg in treated subjects. Exclusion criteria for all subjects were: refusal to participate in the study; presence of ocular pathologies other than glaucoma; evidence or a history of ocular trauma or ocular surgery (except for cataract surgery without complications) in both eyes; the presence of pathologies which may interfere with visual field testing; and the use of drugs which may interfere with visual field testing.

Investigational hypotheses for this study are summarized in Table 1.

**Table 1:** Investigational hypotheses.

1) *Comparison with HFA*	The two devices should provide equivalent results on normal subjects and glaucomatous subjects (average differences within ±2 dB)
2) *Normative database*	A decrease in sensitivity with age of approximately 0.05 dB per year, as well as a decrease with increasing eccentricity should be shown
3) *Precision data*	Average repeatability standard deviations over all grid points should be inferior than 2 dB
4) *Color retinal imaging*	It shall be possible to obtain good quality color images on at least 85% of the subjects presenting with a minimum pupil diameter of 3.0 mm
5) *Examination time*	Average examination time for 24–2 test with 4–2 threshold strategy shall not exceed the time required for a similar test with HFA
6) *Failure rate*	The number of subjects for which the test fails, for various reasons, should be comparable with those failing on HFA

**Fig 1:**
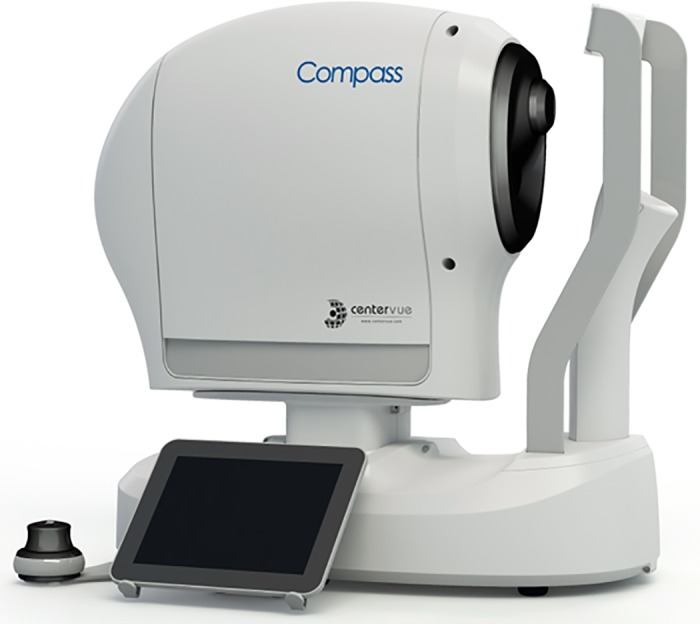
Compass automated fundus perimeter, with tablet and joystick interface.

The Compass unit is shown in Fig. 1. The instrument is 62 cm x 59 cm x 36 cm and includes a perimeter, a scanning ophthalmoscope, an eye tracker to measure fixation and to actively compensate for eye movements during examination.

### Compass procedure

The test is performed in a dark room. After patching the fellow eye, the patient is asked to place his or her forehead against the bar and the chin on the rest. No corrective lenses are required, as the system automatically corrects for spherical aberration and focuses on the retina to compensate for refractive error. Fixation is first evaluated, then VF test is performed with a double control for fixation: a continuous, live infrared image of the retina (available on the operator pad), and an automatic eye tracker. At the end of the procedure, a color image of the retina is collected.

#### Compass as a perimeter

In the current version (software version 0.2), the device evaluates the retinal sensitivity using the following characteristics: 24–2 grid (54 locations spaced by 6 degrees), threshold strategy: 4–2 staircase modeled on accelerated stochastic approximation, stimulus size: Goldmann III, stimulus duration: 200 msec, background luminance: 31.4 asb, maximum luminance: 10,000 asb. As continuous control of fixation is made during the Compass test, a “fixation loss” index (which is present in the HFA) is not recorded.

#### Compass to evaluate fixation stability

Compass acquires live infrared images of the fundus at 25 frames per second. These are processed to identify multiple retinal landmarks and track their movements. By means of this technique, the instrument provides a measure of a patient’s fixation characteristics, with accuracy of about 20 microns (being 17 microns Compass imaging resolution).

Fixation stability was quantified as follows:

- the average X-Y coordinate of all fixation points (P_i_) was calculated (= center of fixation);
- the Cartesian distance (in degrees) of all fixation points from fixation center (D_i_) was determined;
- the percentage of points having distance D_i_ < 1° (P1) was reported as a measure of stability.

**Fig 2:**
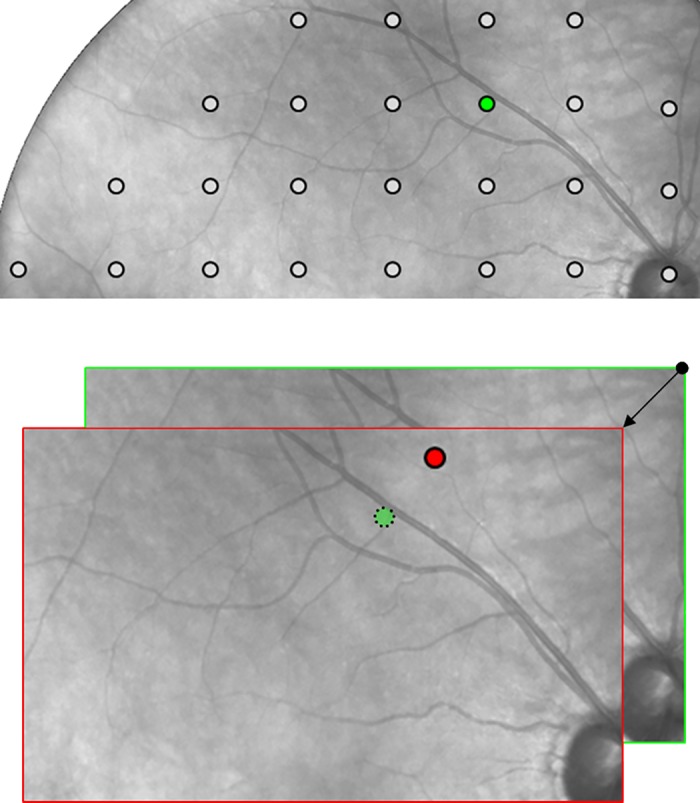
Eye tracking compensation. (A) The green dot shows the expected presentation position on the retina of one of the 24–2 measurement locations. (B) The red dot shows the actual presentation position in presence of a 3-degree eye movement at the time of presentation (see arrow) and in absence of a compensation mechanism: the red dot is where the HFA would actually measure sensitivity, without a chance to time-correlate presentation of this stimulus with the information provided by the gaze tracker. The use of a 25 Hz retinal tracker instead, enables Compass to maintain the expected presentation position even in presence of wide and fast eye movements.

Differently from the HFA (which just records pupil movements), real time retinal tracking allows Compass to actively compensate for eye movements throughout the VF test. This is done by recalculating a stimulus position before it is actually presented, based on the current position of the retina: this compensation mechanism is key to maximize the test reliability, as illustrated in Fig. 2.

Thanks to the retinal tracker, the position of stimulus projection is compensated for minor eye or head movements, i.e. the instrument actively ensures that any stimulus is projected exactly at its programmed retinal location, regardless of fixation losses. In case of major movements, resulting in a lost tracking, projection is paused until the retina is tracked again.

#### Compass as a scanning ophthalmoscope

The Compass instrument collects images of the central retina over a 30° radius field, obtained using infrared or white light sources to illuminate the retina, by means of a confocal setup, using a 5 megapixel (Mp) sensor. The instrument is intended to provide retinal images of sufficient quality for a pupil diameter of 3 mm or more; therefore, no dilation is necessary for most subjects. The instrument does not measure pupil size in the current version.

### Data management

A total of 350 subjects were recruited, with 30 subjects excluded for the following reasons: ten subjects had either Compass or HFA tests outcomes that were clinically judged as unreliable (Compass: 2 cases; HFA: 6 cases, both devices: 2 cases); one subject had an uncertain diagnosis; two subjects had previously undetected co-morbidities (1 with macular edema, 1 with angle-closure glaucoma); and 17 subjects did not complete all parts of the protocol.

Three normal subjects (1.5%) in the Compass dataset and 1 subject (0.5%) in the HFA dataset were classified as outliers (average retinal sensitivity below 3 standard deviations from the mean), and excluded from further analyses. All subjects with glaucoma were considered.

### Statistical analysis

For perimetric comparative analysis, VF sensitivities of left eyes were converted to a stimulus location grid as if they were right eye cases. Locations corresponding with the blind area of the ONH were excluded from the analysis of both instruments. Recorded test times included autofocus to correct spherical aberration, tracking, the VF test, and retinal imaging.

Images were graded as follows. Score-1 (missing picture). Score 0 (insufficient quality): retina not visible or ONH margins not entirely visible. Score 1 (sufficient quality): RNFL partially invisible, or image partially defocused, or image slightly over- or under-exposed. Score 2 (good or excellent quality): fully visible retina, correct focus, and illumination.

Fixation rate was analyzed by considering the percentage of fixation points within 1° from the center of mass of all fixation points recorded during the perimetric test (P1) [13].

Normal and glaucoma subjects were compared with t-test for unpaired data. Coefficient of variation (CV) was obtained by dividing mean repeatability for each group by the respective threshold means in the groups. Bland-Altman plots of the locations within 10° were calculated in order to allow a comparison with the HFA from previously published literature data. [14]

## Results

The main characteristics of the subjects who completed the study are given in Table 2. The relative case distributions per decade of age of the 197 normal subjects whose tests were used to generate the normative database were: 13% (decade 20–29), 8% (30–39); 23% (40–49); 26% (50–59); 20% (60–69); 9% (70–79).

**Table 2:** Characteristics of the study population.

	Total	Normal	Glaucoma
Age, years, mean ± sd (range)	58.5 ± 16.7 (20–86)	50.6 ± 15.2 (20–86)	71.8 ± 8..8 (34–86)
Race, number	320	200	120
Caucasian	319	200	119
Hispanic	1	0	1
Right / Left, number	188 / 132	119 / 81	69 / 51
Female / Male, number	185 / 135	127 / 73	58 / 62
Best-corrected visual acuity	0.93±0.21	0.96±0.07	0.88±0.33

### Comparison with HFA

Mean sensitivity for normal subjects was 28.5 ± 1.6 dB for Compass and 29.5 ± 1.8 dB for HFA and the difference between the two measurements was-1.02 ± 1.55 dB (p<0.001). Mean sensitivity for glaucoma subjects was 21.2 ± 7.1 dB for Compass and 22.2 ± 6.6 dB for HFA and the difference between the two measurements was-1.01 ± 2.81 dB (p<0.001).

**Fig 3:**
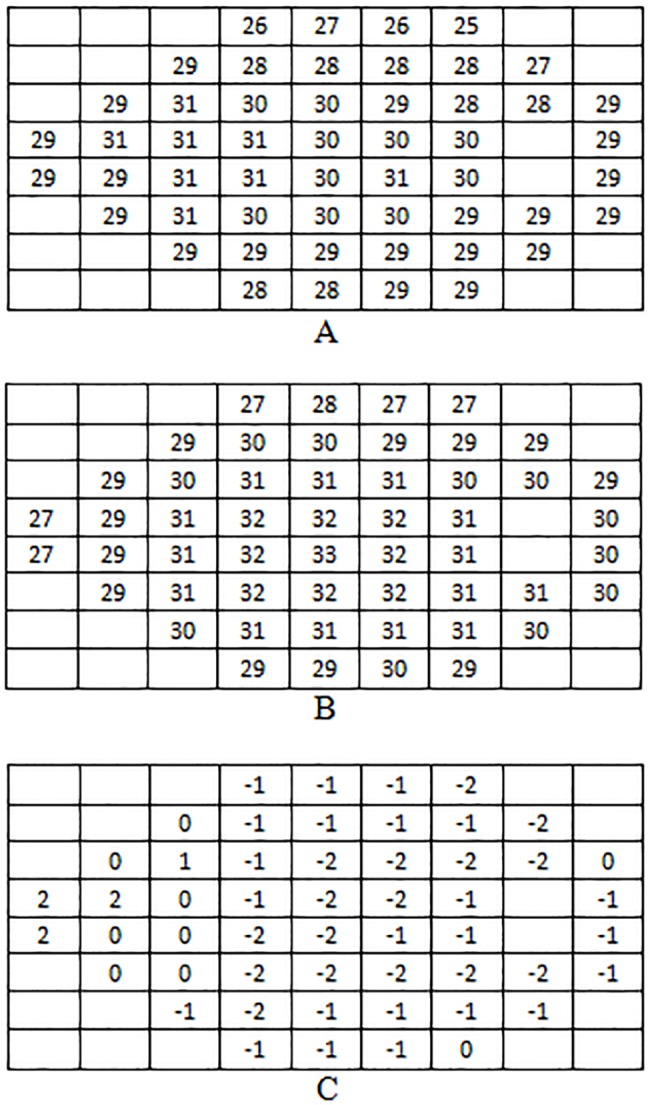
Mean retinal sensitivity at each location for Compass (A), HFA (B), and their difference (C).

Mean retinal sensitivities obtained with both devices at each stimuli location on normal subjects are presented in Fig. 3.

**Fig 4:**
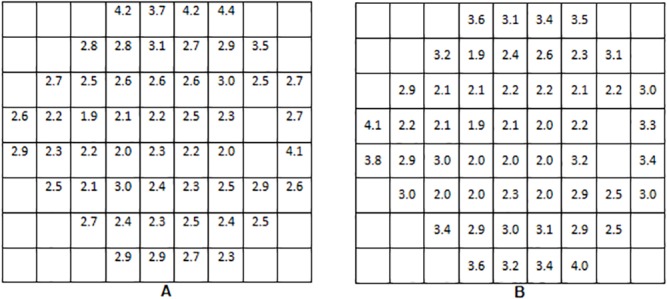
Retinal sensitivity standard deviations at each location in normal group for Compass (A) and HFA (B).

Standard deviations of sensitivities obtained with both devices at each stimuli location on normal subjects are shown in Fig. 4.

### Database

The formulas of global linear regression equations yielding Normal Sensitivity (NS) as a function of age were:

$$\left(\text{Compass}\right)\;\;\;\;\;\;\text{NS}=-0.04*\text{Age}+30.8\;\;\left(p<0{\text{.001, r}}^{2}\text{=0}.17\right)$$

$$\left(\text{HFA}\right)\;\;\;\;\;\;\text{NS}=-0.06*\text{Age}+32.7\;\;\left(\text{p}<0{.001\text{,r}}^{2}=0.28\right)$$

The reduction of retinal sensitivity induced by age in normal subjects was studied additionally on foveal and perifoveal regions (average of fovea and four central locations) and on the central 10°. For Compass, the mean decrease was -0.048 dB / year for the foveal and perifoveal areas, and -0.045 dB / year for the central 10°. For HFA, other published sources report a mean decrease of-0.047 dB / year for the foveal area [15] and of-0.041 dB / year for the central 10° [14].

Independent linear regression equations were derived for each stimuli location, providing an estimate of NS at any grid location, for any specific age. The inferior limit for normal sensitivity at a certain location (x, y) for a subject of a given age was calculated as follows:

INF (x, y, a)=NS (x, y, a)-2SD (x, y, a)

where SD(x, y, a) is the standard deviation of normal sensitivity at location (x, y) for the age group corresponding to a.

A decrease of sensitivity was observed as a function of eccentricity, as expected. This was checked by calculating NS along certain meridians and parallels belonging to the 24–2 grid, for a certain age using the above mentioned point-wise linear regression equations. Such values exhibited a good fit with 2^nd^ order polynomial functions (data not shown), suggesting parabolic behaviors in space, with vertices in the foveal region and decreasing values with increasing eccentricity.

### Compass test-retest variability

A subset of 89 normal subjects (age: 49.6 ± 13.1, range: 20–73 years) and 19 subjects with glaucoma (age: 72.2 ± 8.2, range: 48–81 years) were tested twice with Compass for precision analysis.

For normal subjects, average sensitivity was 29.2 ± 1.5 dB at the first test, and 29.4 ± 1.4 dB at the second test (p = 0.014). For glaucoma patients, average sensitivity was 25.0 ± 4.7 dB at the first test, and 24.5 ± 5.0 dB at the second test (p = 0.011).

**Fig 5:**
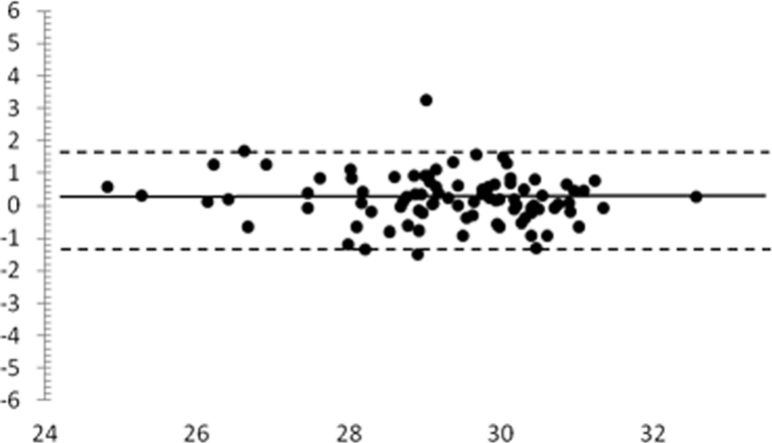
Bland-Altman plot for mean sensitivity (2nd—1st measurement) for normal subjects undergoing test-retest analysis. x, average of mean sensitivity of the two tests (dB). y, difference of mean sensitivity of the two tests (dB).

A Bland-Altman plot for the global sensitivity at test-retest on normal subjects is given on Fig. 5. The horizontal solid line represents the mean difference (+0.2 dB) and the dotted lines represent the 95% limits of agreement between measurements (-1.3 / +1.7 dB).

Repeatability SD for the average sensitivity with the Compass was 1.53 for the Normal group and 1.84 for the Glaucoma group (Table 3). These values correspond to a CV of 0.05 and 0.07 respectively.

**Table 3:** Compass repeatability: SD for Average sensitivity and individual stimuli sensitivities.

Group/Parameter		Repeatability
Normal (N = 89)	**Average Threshold Repeatability SD**	**1.53 dB**
	Maximum Individual Stimulus Repeatability SD	2.20 dB
	95% Percentile Individual Stimulus Repeatability SD	1.98 dB
Glaucoma (N = 19)	**Average Repeatability SD**	**1.84 dB**
	Maximum Individual Stimulus Repeatability SD	2.68 dB
	95% Percentile Individual Stimulus Repeatability SD	2.33 dB

**Fig 6:**
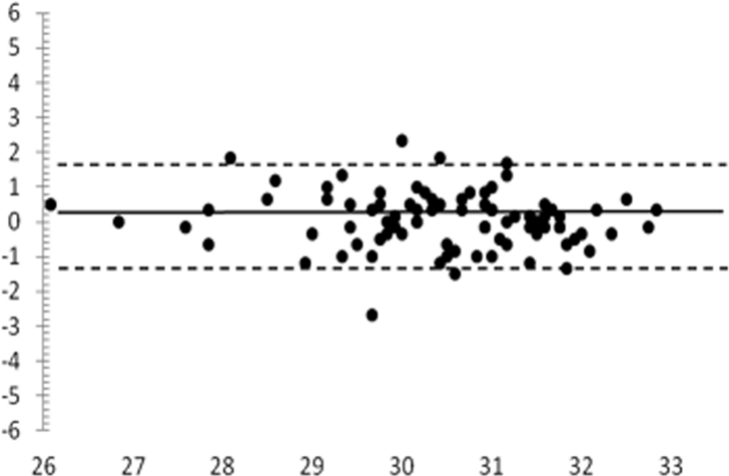
Bland-Altman plots for mean sensitivity of the central 10° (2°– 1° measurement), normal group. x, average of mean sensitivity of the two tests (dB). y, difference of mean sensitivity of the two tests (dB).

A Bland-Altman plot of the locations within 10° is given in Fig. 6: the mean difference was +0.1 dB, and the 95% limits of agreement were-1.5 / +1.6 dB.

### Compass retinal imaging

During perimetry, live infrared images were of adequate quality in 85% of the cases and permitted the visualization of the fundus during perimetry and operation of the retinal tracker in 100% of the cases.

Color images, obtained at the end of the procedure, were evaluated. 93 subjects (29.1%) had a score of-1; 20 (6.3%) of 0; 73 (22.8%) of 1; and 134 (41.9%) of score 2. It was therefore possible to obtain useful (score>0) color images of the central 30° of the retina in approximately 70% of the subjects. Reasons for failure or insufficient quality included: pupil too small; eccentric fixation; subject moved away from device; poor device alignment; image out of focus; dirt on front lens; image too dark / too bright; and eye lids closed.

**Fig 7:**
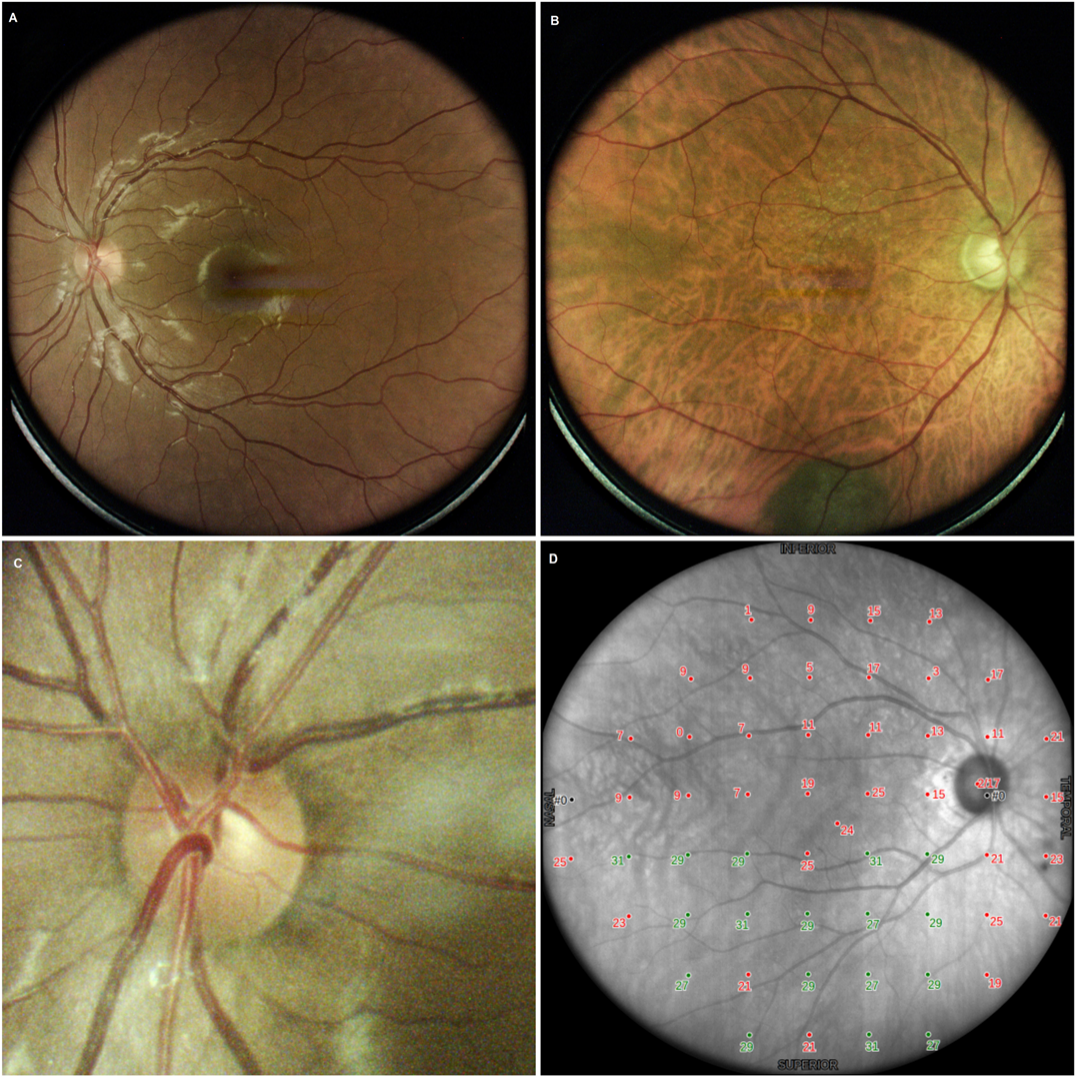
Examples of color images: normal subject (A), glaucoma patient (B), optic nerve head detail (C), infrared image with a sensitivity grid (D).

Examples of fundus images are given in Fig. 7.

### Morphological vs functional data

In order to correlate structural and functional information yielded by Compass, we derived a clinical diagnosis (normal or glaucoma) for all study subjects solely based on the acquired color retinal images, as a method for structural assessment of the optic disc. Then we did the same independently with red-free images, which provide information about the RNFL. The two clinical evaluations were made by an experienced grader and then combined together (“combined morphological diagnosis”), which was compared with the initial one. 319 subjects were considered for this analysis. The agreement with the initial diagnosis was 86.6% for colour images, 82.3% for red-free images, and 87.2% for the combination. Considering the combined diagnosis, the cases of disagreement received a morphological diagnosis of glaucoma in 2.5% of cases, and a morphological diagnosis of normality in 10.2% of cases.

### Compass test time

The average examination time with Compass on all study subjects was 634 ± 96 s per eye. The time was 607 ± 78 s for normal subjects, and 678 ± 108 s for glaucoma patients. The average examination time with HFA (SITA standard) on all study subjects was 309 ± 61 s per eye. The time was 294 ± 44 s for normal subjects, and 358 ± 65 s for glaucoma patients.

### Compass fixation analysis

Fixation rate within the central 1° was 86.6% in normal subjects, and 79.3% in glaucoma patients (5th percentile was 48.9% and 31.0% respectively).

### Usability of the Compass device

Overall, the patients found the test easy to perform. The only adverse event (device-related) was excess lacrimation in about 5% of subjects. Compliance to protocol was excellent.

## Discussion

This pilot study aimed at exploring the diagnostic performance of Compass, a new device for glaucoma diagnosis and follow-up. This instrument is both a perimeter and a scanning ophthalmoscope. It also contains an eye tracker that enables the study of position and stability of fixation (which have been found to be relevant for glaucoma [10,11]) and the active control of locations at which stimuli are presented.

In the management of glaucoma, perimetry plays a key role. As a consequence, the primary objectives of this study were to explore Compass validity as a perimeter in comparison with the currently accepted clinical standard (i.e., the HFA 24° SITA Standard), and to generate a normative database for Compass perimetry.

The instrument fulfilled all the perimetric hypotheses outlined in Table 1 concerning perimetry.

The point-to-point sensitivities calculated over a 24° grid were comparable for Compass and HFA both in normal and glaucoma subjects, as a difference of +1.0 dB was found in both study groups, with HFA measuring higher than Compass. We postulate that Compass provided lower threshold measurements than HFA due to the different thresholding algorithm. In fact SITA standard is known to slightly overestimate threshold vs. full-threshold testing [16]. Another possible source of reduced sensitivity with Compass is fatigue effect; in the current version, perimetric test time is in fact longer than SITA. Yet, the difference was largely within acceptable clinical limits.

The database showed solid characteristics: a trend for a decrease in sensitivity with age of approximately 0.05 dB per year was found, as well as a decrease in sensitivity with increasing eccentricity of the stimulus location. A larger normative database is needed to further differentiate the lower-than-normal results. In particular the sub-category of patients above the age of 70 (where the majority of glaucoma patients occur) is not yet adequately represented.

In addition, precision of the Compass device was good: average SD was 1.53 dB for normal subjects, and 1.84 for glaucoma. In the second examination sensitivity was found to be slightly higher compared to the first one in normal subjects (29.2 vs 29.4 dB), but not in glaucoma patients (25.0 vs 24.5 dB). This may be due to a mild learning effect. A mean test-retest difference of +0.2 dB was found on the whole population; the 95% limits of agreement between overall global sensitivity were-1.3 dB and +1.7 dB.

Precision of the Compass was not directly compared with the HFA in this study. Still, in order to allow comparison with literature data, we built a Bland-Altman plot for central 10° locations, and we found that the Compass 95% limits of agreement were approximately 20% narrower than the HFA’s (-1.5 / +1.6 dB, compared with ∼±2 dB for HFA), suggesting that Compass had a reduced variability compared to HFA [10]. Considering that Compass’ current threshold strategy is less favorable than SITA standard, we postulate that such an advantage may be due to retinal tracking, allowing a precise stimulus presentation on the tested locations.

The quality of infrared images was satisfactory in 85% of the cases and allowed the visualization of the fundus during perimetry and operation of the retinal tracker in 100% of the cases. An infrared image has an advantage to better identify the optic disc and cup margins. Color images could be obtained in 65% of cases; quality was good or excellent in 42%, sufficient in 23%, and insufficient in 9%. In 35% of cases, a color image could not be collected for the causes listed in the Results section; mainly it was due to most patients moving away from the instrument at the end of perimetry or closing their eyelids, which are errors attributed to unfamiliarity of the device by the patient. Further developments of the test procedure are in progress, which should allow for a significant reduction of the percentage of failure, e.g., a vocal message to remind the patient to stay still and open the eyelids as the image is collected. It is also possible to collect retinal images without performing the complete procedure of perimetry + imaging.

ONH and RNFL images confirmed the perimetric diagnosis in about 85% of cases, with no differences between ONH and RNFL. In just 2.5% of cases, imaging suggested a diagnosis of glaucoma. It should be noted that this analysis was based on HFA and not on Compass perimetry (being MD and PSD extrapolated after the creation of a database). These results on the correlation between morphology and function are preliminary and deserve validation on properly designed studies.

Examination time with Compass (634 seconds) was longer than SITA Standard and comparable with full-threshold HFA. The adoption of a database and of different threshold strategies, and the use of different grids will guarantee a significant reduction of test time in the future versions of the software. Of note, the test time with Compass covers a larger span of events (autofocus, VF test with tracker, imaging) than HFA (just perimetry alone). In addition, the time used to refract the patient and align corrective lenses is not considered in the comparison to the HFA.

Finally, failure rate in the study was 1% (four subjects), which was comparable with the HFA failure rate.

Overall, the multi-function Compass device may provide a number of possible advantages over single function perimetric assessment device, such as the HFA.

Major advantages over HFA include the smaller physical size of the instrument, the possibility of having the eye tested without near correction; lower test variability probably due to the increased repeatability in a stimulus projection location by means of live retinal tracking; and the possibility of developing an ad-hoc grid pattern to reduce test duration and improve diagnostic ability.

As a scanning ophthalmoscope, Compass allows continuous retinal monitoring during perimetry and it obtains high-quality images of the central 30° of the retina, with a satisfactory view of optic nerve head, RNFL and retina in 65% of cases. It is anticipated that the percentage of patients who had inadequate color images could be reduced in the next version of the instrument.

The possibility of obtaining images of the posterior pole has several potential advantages:

1. Direct comparison of the optic disc with the perimetric results; which will improve the diagnostic accuracy in those cases with an otherwise normal optic disc.
2. The possibility of an assessment of eventual retinal diseases; this will explain an abnormal test result in patients with a normal optic disc (e.g. myopic atrophy, diabetic retinopathy, macular degeneration, etc.).
3. The possibility of assessing the RNFL and searching for agreement with the perimetric test results (“structure-function relationship”). The ability to image the posterior pole might help to develop a “guided” grid to enhance the diagnostic capability of the functional assessment.

In conclusion, this pilot study explored the characteristics of Compass. We found that, as a perimeter, the instrument had a performance overall similar to HFA; yet, the eye tracker reduced the measurement “noise” and improved data repeatability. As a scanning ophthalmoscope, the instrument provided high-quality images in about 70% of patients, thus allowing a better evaluation of the functional and structural aspects of disease.
